# Bordetella pertussis Can Be Motile and Express Flagellum-Like Structures

**DOI:** 10.1128/mBio.00787-19

**Published:** 2019-05-14

**Authors:** Casandra L. Hoffman, Laura A. Gonyar, Federico Zacca, Federico Sisti, Julieta Fernandez, Ting Wong, F. Heath Damron, Erik L. Hewlett

**Affiliations:** a Division of Infectious Diseases and International Health, Department of Medicine, University of Virginia, Charlottesville, Virginia, USA; b Instituto de Biotecnología y Biología Molecular (IBBM)-CCT-CONICET-La Plata, Departamento de Ciencias Biológicas, Facultad de Ciencias Exactas, Universidad Nacional de La Plata, La Plata, Argentina; c Department of Microbiology, Immunology and Cell Biology, School of Medicine, West Virginia University, Morgantown, West Virginia, USA; The Sanger Institute; University of Michigan; University of Georgia

**Keywords:** *Bordetella*, *Bordetella bronchiseptica*, *Bordetella pertussis*, flagella, flagellar motility, flagellar structure, motility

## Abstract

Bordetella bronchiseptica encodes and expresses a flagellar apparatus. In contrast, Bordetella pertussis, the causative agent of whooping cough, has historically been described as a nonmotile and nonflagellated organism. The previous statements that B. pertussis was a nonmotile organism were consistent with a stop codon located in the flagellar biosynthesis gene, *flhA*, discovered when the B. pertussis Tohama I genome was sequenced and analyzed by Parkhill et al. in 2003 (J. Parkhill, M. Sebaihia, A. Preston, L. D. Murphy, et al., Nat Genet, 35:32–40, 2003, https://doi.org/10.1038/ng1227). The stop codon has subsequently been found in all annotated genomes. Parkhill et al. also showed, however, that B. pertussis contains all genetic material required for flagellar synthesis and function. We and others have determined by various transcriptomic analyses that these flagellar genes are differentially regulated under a variety of B. pertussis growth conditions. In light of these data, we tested for B. pertussis motility and found that both laboratory-adapted strains and clinical isolates can be motile. Upon isolation of motile B. pertussis, we discovered flagellum-like structures on the surface of the bacteria. B. pertussis motility appears to occur primarily in the Bvg(−) phase, consistent with regulation present in B. bronchiseptica. Motility can also be induced by the presence of fetal bovine serum. These observations demonstrate that B. pertussis can express flagellum-like structures, and although it remains to be determined if B. pertussis expresses flagella during infection or if motility and/or flagella play roles during the cycle of infection and transmission, it is clear that these data warrant further investigation.

## Abstract (precis)

This report provides evidence for motility and expression of flagella by B. pertussis, a bacterium that has been reported as nonmotile since it was first isolated and studied. As with B. bronchiseptica, B. pertussis cells can express and assemble a flagellum-like structure on their surface, which in other organisms has been implicated in several important processes that occur *in vivo*. The discovery that B. pertussis is motile raises many questions, including those regarding the mechanisms of regulation for flagellar gene and protein expression and, importantly, the role of flagella during infection. This novel observation provides a foundation for further study of *Bordetella* flagella and motility in the contexts of infection and transmission.

## OBSERVATION

Bordetella pertussis evolved from Bordetella bronchiseptica, which encodes and expresses the proteins for a functional flagellum. Sequencing of B. pertussis Tohama I and B. bronchiseptica RB50 by Parkhill et al. revealed that genes for flagellar biosynthesis and functions are present in both genomes (1–3). There was, however, a stop codon located 1,313 bases into *flhA* (total gene, 2,119 bases) of B. pertussis. FlhA is a transmembrane, type III secretion protein that serves as docking site for Fli(X) ATPases and FliC filaments. FlhA is responsible for the export of FliC filaments for flagellar tail assembly (4) and is described as one of the 24 core proteins essential for flagellar assembly (5). Thus, a stop codon in *flhA* appeared consistent with B. pertussis being nonflagellated and nonmotile (6).

Information about regulation and relevance of *Bordetella* motility and flagellar gene and protein expression is largely limited to work with B. bronchiseptica. Akerly et al. showed that B. bronchiseptica flagellar expression and motility are controlled by the BvgAS two-component system (7, 8), which modulates among the virulent Bvg(+) phase, intermediate Bvg(i) phase, and avirulent Bvg(−) phase. Flagellar gene and protein expression and the motile phenotype occur primarily in the Bvg(−) phase (8). These findings have raised questions about relevance of motility and flagellar expression during infection, as these phenotypes are associated with the avirulent Bvg(−) phase. Recently, van Beek et al. found that within the mouse respiratory tract, B. pertussis expresses Bvg(−) genes, including those from the flagellar operon (9), and Bvg(−) B. pertussis strains have been isolated from patients during infection (10). In addition, flagellar expression and motility appear to be important for virulence phenotypes. B. bronchiseptica motility is required to reach intracellular niches within the host; flagella are involved in both motility and adherence to biotic and abiotic surfaces (6, 11–13). These data suggest that during the Bvg(−) phase, flagellar expression and motility may have roles in infection and/or transmission.

### B. pertussis can be motile.

Several data sets have demonstrated differential regulation of B. pertussis genes associated with assembly and function of flagella. Specifically, Barbier et al. compared a wild-type (WT) strain (UT25) to the UT25 Δ*rseA* mutant (14). In UT25 Δ*rseA*, RpoE functions were increased, and surprisingly, genes associated with flagellar assembly and function were increased between 1.5- and 22-fold (15). Additionally, expression of flagellar genes has been observed during mouse infection (9, 16). Based on these data sets, we tested the hypothesis that B. pertussis produces flagellar proteins, enabling B. pertussis motility.

**FIG 1:**
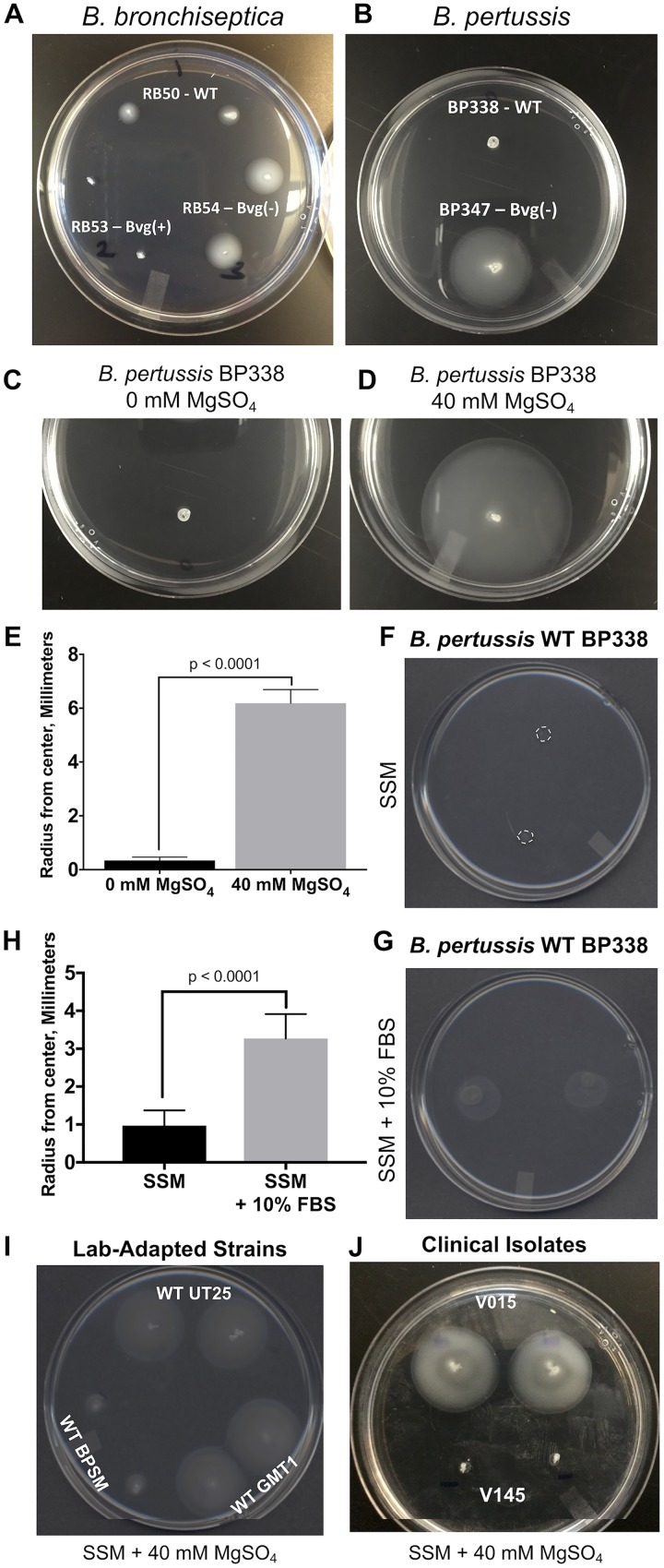
B. bronchiseptica and B. pertussis are motile in the Bvg(−) phase. Bacteria were grown overnight as shaking cultures in Stainer-Scholte Medium (SSM) and diluted to an optical density at 600 nm (OD_600_) of 0.800. Two microliters of diluted cultures was stabbed into 0.4% SSM agar plates. B. bronchiseptica strains were grown for 24 h at 37°C and ambient CO_2_ levels, B. pertussis strains were grown for 72 h under the same conditions. (A) B. bronchiseptica WT RB50, Bvg(−) RB54, and Bvg(+) RB53 were tested for motility. (B) B. pertussis WT BP338 and Bvg(−) BP347 were tested for motility. The B. pertussis motility zone increases when the bacteria are modulated to the Bvg(−) phase with 40 mM MgSO_4_. B. pertussis WT BP338 cells were grown overnight as shaking cultures in SSM and diluted to an OD_600_ of 0.800. Two microliters of diluted cultures was stabbed into 0.4% SSM agar plates. B. pertussis strains were grown for 72 h at 37°C at ambient CO_2_ levels. (C and D) Representative images of BP338 grown without (C) and with (D) 40 mM MgSO_4_. The experiment was repeated 6 times, and the radius was quantitated each time. WT BP338 has dashed outlines in panel F to better show the radius of the spreading zone. (E) The mean radius with standard deviation was graphed for each condition (±40 mM MgSO_4_). *P* < 0.0001. Serum increases B. pertussis motility. (F and G) Representative images of BP338 grown without (F) and with (G) 10% fetal bovine serum (FBS) in motility agar. The experiment was repeated 6 times, and the radius was quantitated each time. (H) The mean radius with standard deviation was graphed for each condition (±10% FBS). *P* < 0.0001. Lab-adapted and clinical isolates demonstrate a motile phenotype under Bvg(−)-modulated conditions. Bacteria were grown overnight as shaking cultures in SSM and diluted to an OD_600_ of 0.800. Two microliters of diluted cultures was stabbed into motility agar plates containing 0.4% SSM plus 40 mM MgSO_4_. B. pertussis strains were grown for 72 h at 37°C at ambient CO_2_ levels. (I) WT UT25, WT BPSM, and GMT1 (J) Clinical isolates V015 and V145.

We examined B. pertussis for motility during growth in soft agar, as previously described for bordetellae, using B. bronchiseptica WT strain RB50, Bvg(+) RB53, and Bvg(−) RB54 as controls (8). As expected, from previous observations, WT RB50 is motile, Bvg(+) RB53 is nonmotile, and Bvg(−) RB54 is motile at 24 h when grown at 37°C (Fig. 1A). B. pertussis WT BP338 and a Bvg(−) mutant (Tn5::*bvgS*) BP347 (17) were stabbed into motility agar, grown at 37°C, and observed over the course of 72 h. Although the WT BP338 strain was nonmotile, the Bvg(−) mutant BP347 was motile at 72 h (Fig. 1B). In several experiments, we observed that B. pertussis WT BP338 and another lab-adapted B. pertussis WT strain (BPSM) could become motile without additional manipulation, but this did not occur consistently (in <15% of experiments). An example is included in Fig. S1 in the supplemental material. We hypothesized that when the B. pertussis WT strain becomes motile, it is due to either phase variation to the Bvg(−) phase or a genetic mutation that results in Bvg(−) mutants that dominate and spread. To test for Bvg(−) mutants, motile bacteria from outer edges of WT BP338 spreading zones were isolated and replated on fresh plates. This yielded both Bvg(+) and Bvg(−) colonies, based upon colony size and hemolysis on Bordet-Gengou (BG) blood agar plates. Bvg(+) and Bvg(−) colonies were then isolated by replating the individual colonies on fresh BG blood agar plates. These isolated bacterial populations were then used to grow overnight liquid cultures, and motility assays were completed. This had no effect on the motile phenotypes of these bacteria. (The motile phenotype was still variable.)

10.1128/mBio.00787-19.2 FIG S1 Images of two technical replicate plates from one experiment, demonstrating that B. pertussis WT strains can become motile under nonmodulated conditions. The indicated B. pertussis strains [WT BP338, Bvg(−) BP347, and WT BPSM] were grown overnight as shaking cultures in SSM and diluted to an OD_600_ of 0.800. Two microliters of diluted liquid cultures was stabbed into 0.4% SSM agar plates without MgSO_4_ or 10% fetal bovine serum. Importantly, all plates were prepared at the same time. Plates were then placed at 37°C and ambient CO_2_ levels for 72 h. Plates 1 and 2 both show the Bvg(−) strain is motile (to various extents). Plate 2 shows that WT BP338 and WT BPSM become motile under the same growth conditions. It is unclear what causes WT strains to become motile under nonmodulated conditions. This did not occur frequently enough during experiments to quantify, but may provide a clue as to which specific signal or signals induce motility. Download FIG S1, PDF file, 1.0 MB. Copyright © 2019 Hoffman et al. 2019 Hoffman et al. This content is distributed under the terms of the Creative Commons Attribution 4.0 International license.

To confirm Bvg regulation of motility, plates were supplemented with 40 mM MgSO_4_ to elicit chemical modulation of B. pertussis to the Bvg(−) phase. After 72 h, the radius of the motility zone was recorded. Representative images from six experiments show that MgSO_4_ increased the B. pertussis WT BP338 zone of motility (Fig. 1C and D). Data from these six experiments were combined (Fig. 1E) and show that the mean radius of the B. pertussis BP338 motility zone is increased in the presence of 40 mM MgSO_4_. Due to reports of serum increasing virulence characteristics, independent of BvgAS activation (18), we also added 10% fetal bovine serum (FBS) to the motility agar plates, ensuring that the agar concentration remained at 0.4%. The presence of serum significantly increased motility in B. pertussis (Fig. 1F to H).

To determine if the above data are indicative of a general phenomenon, we tested lab-adapted and clinical isolates and found that some, but not all, were consistently motile. This was the case for WT BP338 and Bvg(−) mutant BP437. It is not yet clear as to why B. pertussis is not always motile under motility-promoting conditions. Table 1 describes all strains tested and their motility phenotype in the presence of 40 mM MgSO_4_ (+, motile; −, nonmotile). Representative examples of strains demonstrating motility in these assays when modulated to the Bvg(−) phase are presented in Fig. 1 (lab-adapted strains in panel I and clinical isolates in panel J [for more information, see Table 1]). The observed B. pertussis spreading occurred within the agar layer, a feature indicative of swimming motility, which, in other bacteria, is flagellum dependent (19). Upon isolation of motile B. pertussis from the outer edge of motility halo at 48 h, a video recording of live bacteria (×1,000 magnification) shows rapid movement across the field of view, also indicative of swimming motility (see Movie S1 in the supplemental material).

**TABLE 1:** B. pertussis motility under Bvg(−)-modulating conditions^*a*^

Strain	Motile phenotype with 40 mM MgSO_4_
WT BP338 (Tohama I)	+
Bvg(−) BP347	+
WT Bpe60 (Tohama I)	+
WT BP536 (Tohama I)	–
WT UT25	+
WT BPSM (Tohama I)	+
WT GMT1	+
Clinical isolates	
V015	+
V145	–
V235	–
UVA009	–
UVA010	+
UVA015	+
UVA018	–
UVA052	+
UVA062	–
UVA145	–
UVA150	+
UVA175	+
UVA190	+
UVA194	–
UVA198	–

a Strains from different isogenic backgrounds and clinical isolates were grown as described for the motility agar assay. Motility, measured by outward spreading from the point of inoculation in the agar, was determined to be positive (+) if the strain was consistently (>80%) motile under modulated conditions (40 mM MgSO_4_). If the strain was not consistently motile, the motility phenotype was determined to be negative (−). Experiments were repeated 5 times; the strains were tested in duplicate in each experiment.

10.1128/mBio.00787-19.3 MOVIE S1 Video recording **(**1,000× magnification) of live B. pertussis WT BP338 bacteria, isolated from the outer edge of the motility halo at 48 h of growth on motility agar plates containing 40 mM MgSO_4_. The video shows rapid movement of several individual bacteria across the field of view. Download Movie S1, MOV file, 8.8 MB. Copyright © 2019 Hoffman et al. 2019 Hoffman et al. This content is distributed under the terms of the Creative Commons Attribution 4.0 International license.

### Bordetella pertussis cells can express flagellum-like structures on their surface.

**FIG 2:**
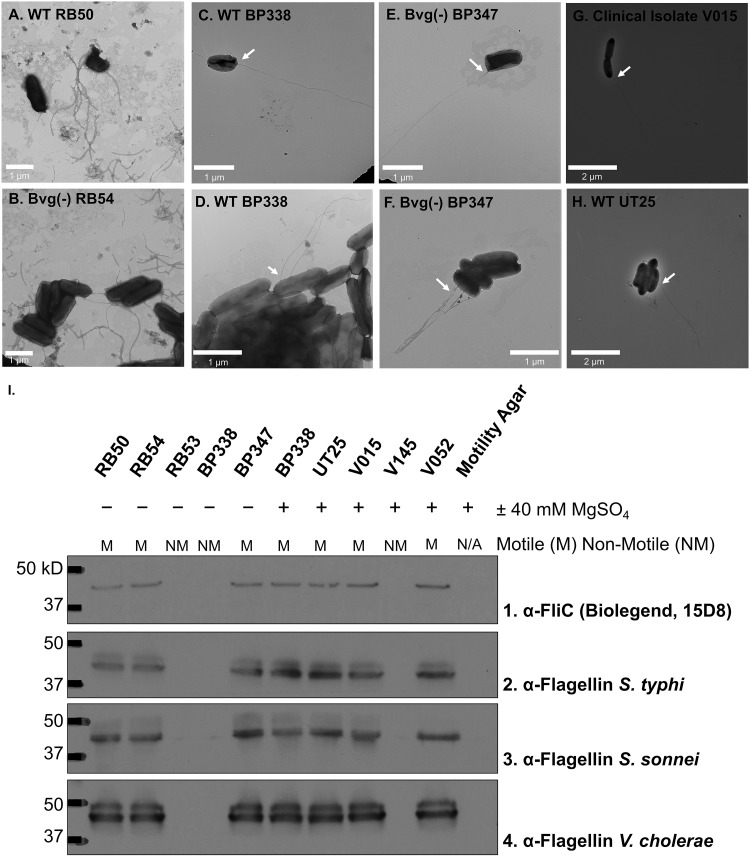
Negative-stain TEM of B. bronchiseptica and B. pertussis shows flagellar structures on bacterial surface. Presumably motile B. bronchiseptica strains were isolated from the outer edges of the spreading zones in 0.4% SSM agar plates plus 40 mM MgSO_4_. (A) WT RB50, (B) Bvg(−) RB54, (C and D) lab-adapted WT BP338, (E and F) the BP338-derived Bvg(−) BP347, (G) recent clinical isolate V015, and (H) lab-adapted WT UT25 were isolated for negative-stain TEM as described in the methods and imaged with a JEOL 1230 transmission electron microscope. Representative images of flagellated bacteria were selected; not all observed bacteria were flagellated. The experiment was repeated 3 times for WT RB50, Bvg(−) RB54, WT BP338, and BP338-derived Bvg(−) BP347. The experiment was repeated twice for clinical isolate V015 and WT UT25. (I) Western blot analysis of flagellin protein expression of motile B. bronchiseptica and B. pertussis strains. Presumably motile B. pertussis strains were isolated from the outer edges of the spreading zones in plates containing 0.4% SSM agar plus 40 mM MgSO_4_. Samples were prepared as described in the methods. Nitrocellulose membranes were probed with a variety of flagellin antibodies. (I, row 1) BioLegend monoclonal anti-FliC antibody. (I, row 2) Anti-*Salmonella* Typhi flagellin antibody. (I, row 3) Anti-Shigella sonnei flagellin antibody. (I, row 4) Anti*-*Vibrio cholerae flagellin antibody. Noncommercial antibodies were obtained from Jorge Giron and used previously to characterize *Shigella* flagella.

B. bronchiseptica motility is mediated by flagella, as B. bronchiseptica Δ*flaA* mutants are nonmotile (7, 8). Although we have not shown that B. pertussis motility is flagellum dependent, in light of our data, we examined motile B. pertussis from soft agar plates containing 40 mM MgSO_4_ for the presence of flagella. Bacteria were isolated from the outer edges of the spreading zones and prepared for negative-stain transmission electron microscopy (TEM) using methods adapted from Akerly et al. (8). B. bronchiseptica strains were flagellated (RB50 in Fig. 2A and RB54 in Fig. 2B) and had multiple flagella per bacterium, as previously described. The B. pertussis BP338 WT and the BP347 Bvg(−) mutant expressed thin flagellar structures on their surfaces, most frequently only one flagellum per bacterium. There were no differences in the number or frequency of flagellated WT BP338 and Bvg(−) BP347, but only approximately 23% of total observed bacteria were flagellated (representative images of flagellated BP338 and BP347 are shown in Fig. 2C to F). The lab-adapted strain B. pertussis UT25 and the clinical isolate B. pertussis V015 had flagellum-like structures on their surfaces (Fig. 2G and H).

To confirm that B. pertussis is able to produce flagellin, we tested B. pertussis isolated from motility agar for reactivity with monoclonal antibody 15D8, which recognizes flagellin (FliC) from B. bronchiseptica. All motile strains react with the monoclonal FliC antibody, and nonmotile strains do not. With this method, although flagellin is clearly being produced, it is possible that the reacting flagellin was intracellular and not exclusively extracellular (from an assembled flagellum). Because of the low frequency of flagellated B. pertussis and the single flagellum per bacterium, we used whole bacteria to test for the presence of flagellin. Using previously described flagellum purification methods to shear flagellar structures from the bacterial surface, we were unable to detect flagellin protein.

A panel of antibodies raised against flagellins from individual bacterial species, Salmonella enterica serovar Typhi (Fig. 2I, row 2), Shigella sonnei (Fig. 2I, row 3), and Vibrio cholerae (Fig. 2I, row 4) (20) recognized an ∼40-kDa band in all motile B. pertussis strains, providing evidence that these B. pertussis strains express flagellin protein. These data, taken with the negative-stain TEM images, confirm that motile B. pertussis cells are able to display flagellum-like structures on their surfaces and express flagellin protein that is immunologically comparable to that from B. bronchiseptica and other Enterobacteriaceae.

Despite microbiological literature stating that “B. pertussis is a nonmotile organism” (21), we have demonstrated that B. pertussis can be motile and express flagella. Several lab-adapted strains and clinical isolates are motile, and B. pertussis motility is enhanced in the Bvg(−) phase. These motile strains express flagellum-like structures and flagellin protein, as verified by negative-stain TEM and Western blotting. Regardless of our inability to detect, specifically, flagellin protein that has been exported to the bacterial surface, we still observe motile bacteria and believe that B. pertussis motility is the major phenomenon described here. These data represent novel and unanticipated observations, which raise many questions to be answered in future studies.

While the genomes of B. pertussis encode the genetic material for a functional flagellar apparatus, existing dogma and the stop codon in *flhA*, which would be expected to preclude expression of FlhA, have been major disincentives to investigate motility in this species. The ability of B. pertussis to express flagellum-like structures raises an important question: how does B. pertussis overcome this apparent impediment in order to make functional flagella? In some bacteria, there are mechanisms for “antitermination” (bypassing the stop codon) (22). Alternatively, BP2261 (BcrD [putative type III secretion apparatus protein]), which has sequence homology to FlhA of B. pertussis (55% homologous) and FlhA of P. aeruginosa (59% homologous), is encoded in the B. pertussis genome. It is possible that BcrD can substitute for FlhA, enabling B. pertussis to form a functional flagellum. Future studies should explore the possible roles of alternative mechanisms to enable motility.

Another explanation for the inconsistency of motility in B. pertussis may be a low efficiency of FlhA (or a substitute) in transporting flagellar components. The predicted stop codon in the *flhA* gene is located at base 1313, potentially yielding an FlhA lacking the C-terminal domain, which in other bacterial species is involved in the export process (23). A Δ*flhA Salmonella* mutant, complemented with FlhA lacking the C-terminal domain, did not assemble a functional flagellum on its surface. However, when the Δ*flhA* bacteria were complemented with *flhA* lacking only certain portions of the C-terminal domain, this resulted in complementation at extended incubation times, suggesting the C-terminal domain is necessary for efficient flagellar assembly (24). B. pertussis FlhA may lack only a portion of the C-terminal domain, resulting in inefficient export and flagellar assembly.

These data do not address the relevance of flagellar expression or motility for virulence and pathogenicity, due to these phenotypes occurring in the Bvg(−) phase. However, Karataev et al. and Medkova et al. have shown recently that Bvg(−) organisms are present in the upper respiratory tracts of infected humans and mice (10, 25). Furthermore, expression of flagellar genes has been demonstrated *in vivo* in mice. van Beek et al. and Wong et al. have identified, by microarray and transcriptome sequencing (RNA-seq), flagellar gene transcripts from the mouse respiratory tract (9, 16). These data demonstrating that B. pertussis can express a flagellum-like structure and be motile, coupled with observation of Bvg(−) organisms and flagellar genes *in vivo*, should prompt the exploration of B. pertussis motility and the mechanisms that govern flagellar expression.

For detailed methods, see Text S1 in the supplemental material.

10.1128/mBio.00787-19.1 TEXT S1 Supplemental materials and methods. Shown are methods used for all described assays and specific materials and original citations for B. pertussis and B. bronchiseptica strains. Download Text S1, DOCX file, 0.02 MB. Copyright © 2019 Hoffman et al. 2019 Hoffman et al. This content is distributed under the terms of the Creative Commons Attribution 4.0 International license.
