# Supplementary figures and images for: Bordetella pertussis Can Be Motile and Express Flagellum-Like Structures
Source: mBio. 2019 May 14;10(3):e00787-19. doi: 10.1128/mBio.00787-19 (PMC6520453; doi:10.1128/mBio.00787-19)

Plate 1

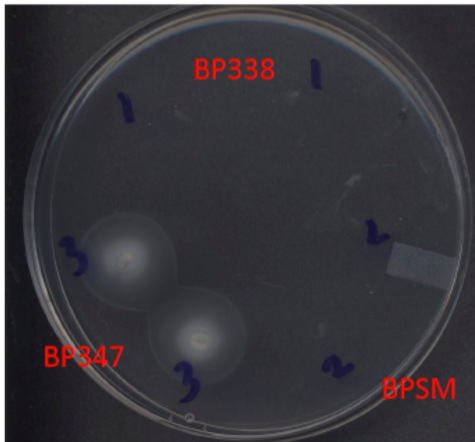

Plate 2

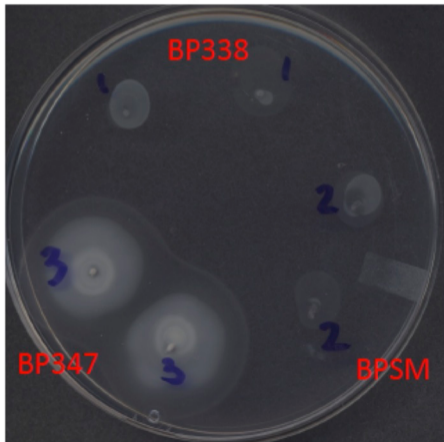

Supplement: FIG S1 [file mBio.00787-19-sf001.pdf]
