# Supplementary material for: Bordetella pertussis Can Be Motile and Express Flagellum-Like Structures
Source: mBio. 2019 May 14;10(3):e00787-19. doi: 10.1128/mBio.00787-19 (PMC6520453; doi:10.1128/mBio.00787-19)
Supplement: TEXT S1 [file mBio.00787-19-s0001.docx]

**Materials and Methods.** **(i) Bacterial Strains and Growth Conditions:** *B. pertussis* WT (wild type) BP338 (Tohama I) (1); Bvg(-) BP347 (TN5::*bvgS* mutant derived from BP338) (1); WT BPSM (Tohama I) (2); WT UT25 (3, 4); WT Bpe60 (5, originally isolated by Duncan Maskell, Sanger Center, Japan 1954); WT BP536 (6); clinical isolate V235 (originated in Hewlett Lab, isolated from University of Virginia patient); clinical isolates UVA009, UVA 010, UVA015, UVA018, UVA052, UVA062, UVA145, UVA150, UVA190, UVA194, UVA198 (obtained from Virginia State Labs and characterized in the Eby/Hewlett lab) were grown on Bordet-Gengou (BG) agar (Gibco) supplemented with 15% defibrinated sheep blood (Cocalico) for 48 hours at 37ºC. The same growth media were used for *B. bronchiseptica* strains, WT RB50 (7); Bvg(-) RB54 (7); and Bvg(+) RB53 (7). *B. bronchiseptica* were grown at 37ºC for 24 hours. After growth on BG plates, bacteria were transferred to 10 mL liquid culture in modified synthetic Stainer-Scholte liquid medium (SSM) with proline and supplement, and grown for 20 hours at 37ºC, shaking at 150 RPM. **(ii) Motility Assay:** The motility assay was modeled after the assay described for *B. bronchiseptica* (8). Motility agar plates were made fresh before each assay from SSM with proline and supplement; the final agar concentration was 0.4% (Hoefer, Inc. GR140500). Each motility agar plate had 15 mL of media added, plates were allowed to set for 2 hours before bacteria were stabbed into the agar. *B. pertussis* were grown on BG, and passaged once in SSM for 20 hours. Bacteria were then diluted to an OD_600_ of 0.800 and 2 µL of the suspension was stabbed into SSM motility agar plates. *B. pertussis* were grown for 72 hours at 37ºC under the various conditions for the motility assay, *B. bronchiseptica* were grown for 24 hours at 37ºC under the various conditions. Importantly, the observations that *B. pertussis* are motile and that motility increases in the presence of 40 mM MgSO_4_, were confirmed by independent labs, and are not resultant of a contaminant. **(iii) Microscopy:** *B. pertussis* WT BP338 bacteria from outer limits of halos from 40 mM MgSO_4_ motility plates were collected 48 hours post inoculation at 37°C. Bacteria were diluted in phosphate buffer and observed using a Nikon Eclipse E200 microscope with 1000X augment. **(iv) Negative Stain TEM:** Motile *B. pertussis* and *B. bronchiseptica* were isolated from motility agar plates using a Fisherbrand Polyester-Tipped Applicator (Cat No. 23-400-122), and swabbed into 1mL filter sterilized, deionized water. Remaining agar chunks were removed by slow speed 2,000-RPM centrifugation. Bacteria solution was centrifuged at 10,000-RPM for 10 minutes to pellet bacteria, which were resuspended in 2.5% gluteraldehyde and fixed for one hour. Bacteria were added to 200 mesh Formvar, copper Transmission Electron Microscopy grids (Ted Pella, Inc. 01700-F), excess liquid was dabbed away. Grids were stained with 2% phophsotungstic acid (pH 7.0) (*79690 SIGMA-ALDRICH*) and excess liquid was dabbed away. Negative-stained *B. bronchiseptica* and *B. pertussis* were imaged with the JEOL 1230 Transmission Electron Microscope. **(v) Western blot analysis for flagellin:** Motile *B. pertussis* and *B. bronchiseptica* were isolated from motility agar plates using a Fisherbrand Polyester-Tipped Applicator (Cat No. 23-400-122), and swabbed into 0.5 mL filter sterilized, deionized water. Remaining agar chunks were removed by slow speed 2,000-RPM centrifugation. Samples were OD_600_-matched to standardize bacterial number. Sample buffer was added and samples were boiled 5 minutes before loading 35 µL to each well of a 12 well, SDS PAGE 10% gel (Criterion TGX, 10% 12+2 well, item no. 5671033). Proteins were transferred at 4ºC to nitrocellulose (MILLIPORE Immobilon-FL, cat no. IPFL00010) overnight at 20V. Blots were probed with flagellin antibodies as described in results and figure legends.

**References**

1. Weiss AA, Hewlett EL, Myers GA, Falkow S. Tn5-induced mutations affecting virulence factors of bordetella pertussis. *Infect Immun.* 1983 Oct; 42(1): 33-41. PMCID: PMC264520.

2. Antoine, R and Locht, C. Roles of the Disulfide Bond and the Carboxy-Terminal Region of the S1 Subunit in the Assembly and Biosynthesis of Pertussis Toxin. *Infect Immun.* 1990 June; 58(6):1518-26. PMCID: PMC258665.

3. Hanawa T, Yonezawa H, Kawakami H, Kamiya S, Armstrong SK. Role of bordetella pertussis RseA in the cell envelope stress response and adenylate cyclase toxin release. *Pathog Dis.* 2013 Jul 2.

4. Barbier M, Boehm DT, Sen-Kilic E, Bonnin C, Pinheiro T, Hoffman C, Gray M, Hewlett E, Damron FH. Modulation of pertussis and adenylate cyclase toxins by sigma factor RpoE in bordetella pertussis. *Infect Immun.* 2016 Dec 29; 85(1): 10.1128/IAI.00565-16. Print 2017 Jan. PMCID: PMC5203664.

5. Gogol EB, Cummings CA, Burns RC, Relman DA. Phase variation and microevolution at homopolymeric tracts in bordetella pertussis. *BMC Genomics.* 2007 May 17; 8: 122. PMCID: PMC1891110.

6. Quandt J, Hynes MF. Versatile suicide vectors which allow direct selection for gene replacement in gram-negative bacteria. *Gene.* 1993 May 15; 127(1): 15-21.

7. Cotter PA, Miller JF. BvgAS-mediated signal transduction: Analysis of phase-locked regulatory mutants of bordetella bronchiseptica in a rabbit model. *Infect Immun.* 1994 Aug; 62(8): 3381-3390. PMCID: PMC302969.

8. Akerley BJ, Monack DM, Falkow S, Miller JF. The bvgAS locus negatively controls motility and synthesis of flagella in bordetella bronchiseptica. *J Bacteriol.* 1992 Feb; 174(3): 980-990. PMCID: PMC206178.
